# The efficacy and sustainability of a reduced risankizumab dose after achievement of low disease activity of psoriasis: a 24-week study^؆^

**DOI:** 10.1016/j.abd.2026.501335

**Published:** 2026-04-29

**Authors:** Gizem Pehlivan Ulutaş, Tuğba Özkök Akbulut, Algün Polat Ekinci

**Affiliations:** a Department of Dermatology and Venereology, Haseki Training and Research Hospital, University of Health Sciences, Istanbul, Turkey; b Department of Dermatology and Venereology, Liv Hospital Ulus, Istanbul, Turkey; c Department of Dermatology and Venereology, Faculty of Medicine, Istanbul University, Istanbul, Turkey

Dear Editor,

Biologic drugs have significantly transformed the management of psoriasis due to their high efficacy, enabling many patients to achieve complete or near-complete clearance.^1^ While sustained treatment is recommended to maintain efficacy, continuous administration at the standard dosing regimen may lead to overtreatment for some patients.^2^ Dose reductions may reduce the cumulative biologic drug exposure, potential adverse effects, and healthcare costs.^3^, ^4^, ^5^, ^6^ On the other hand, this approach requires individualized treatment with close monitoring for relapses.

Studies that report dose reductions primarily focus on TNF-alpha and IL-12/23 inhibitors.^3^, ^4^, ^5^, ^6^, ^7^ Data on IL-23 inhibitors remain limited.^8^, ^9^ Risankizumab, an IL-23 inhibitor, is recommended for psoriasis at a dose of 150 mg subcutaneously at weeks- 0 and -4 and every 12-weeks thereafter. Our study aimed to evaluate the efficacy and sustainability of a reduced risankizumab dose (75 mg every 12 weeks) in patients achieving complete or near-complete clearance, and to explore factors associated with successful outcomes.

This retrospective study was conducted at a tertiary psoriasis clinic. We reviewed the patients treated with risankizumab between 2021 and 2024. The treatment was initiated according to the recommended standard dosing scheme. Eligibility for dose reduction required minimal disease activity after a minimum of 52-weeks of treatment, defined as absolute PASI (Psoriasis Area and Severity Index) ≤3 and inactive psoriatic arthritis for the preceding 24-weeks. With patient consent, the maintenance dose was reduced to 75 mg every 12-weeks.

Patients with flares (PASI score >3 and/or exacerbation of psoriatic arthritis) were escalated back to standard dosing or switched to another biologic. Demographic and clinical data included age, sex, Body Mass Index (BMI), involvement of special areas, presence of psoriatic arthritis, comorbidities, previous systemic treatments, and PASI scores at baseline, week-16, and week-52 of the recommended risankizumab dose, and at dose reduction, weeks-12 and -24 following reduced-dose maintenance treatment. PASI scores and psoriatic arthritis activity were recorded at week-12 in patients who were switched back to the standard risankizumab dose due to relapse of skin or joint symptoms.

Continuous variables are expressed as mean ± SD, categorical variables as n (%), and compared using appropriate tests. A two-tailed p < 0.05 was considered significant. The study received approval from the institutional review board (approval number: 72‒2024).

A total of 21 patients (14 males, 7 females; mean age 47.5±16.7 years) were included; 7 (33.3%) had concomitant psoriatic arthritis. The characteristics of the patients are summarized in Table 1.

**Table 1:** Demographic and clinical characteristics of patients and prior treatments.

	Total group (n = 21)
Age, mean ± SD	47.5 ± 16.7
Gender, n (%): male/female	14 (66.7) / 7 (33.3)
Body mass index, mean ± SD	27.1 ± 3.7
Psoriatic arthritis, n (%)	7 (33.3)
Comorbidities	n (%)
Dyslipidemia	4 (19.0)
Obesity	4 (19.0)
Coronary artery disease	3 (14.3)
Hypertension	3 (14.3)
Hepatosteatosis	2 (9.5)
Benign prostatic hyperplasia	2 (9.5)
Diabetes mellitus	1 (4.8)
Chronic hepatitis B infection	1 (4.8)
Pilocytic astrocytoma	1 (4.8)
Special area involvement	n (%)
Nails	14 (66.7)
Scalp	12 (57.1)
Intertriginous	9 (42.9)
Palmoplantar	2 (9.5)
Previous systemic treatments	n (%)
Methotrexate	18 (85.7)
Acitretin	8 (38.1)
Cyclosporine	5 (23.8)
Narrowband UVB	8 (38.1)
Etanercept	1 (4.8)
Adalimumab	1 (4.8)
Ustekinumab	2 (9.5)
Ixekizumab	1 (4.8)

SD, Standard Deviation.

The mean PASI score at baseline was 14.3±6.2 (range 7.7–38.6). Dose reductions were made at a mean of 75.4-weeks from baseline (range 52–100 weeks), at which time the mean PASI score was 0.6 (range 0‒2.4), with 47.6% of the patients having complete clearance. The PASI scores of the patients before and after dose reduction are summarized in Table 2.

**Table 2:** PASI scores before and after dose reduction with comparison between groups of sustained efficacy and relapse.

	Total group (n = 21)	Relapse group (n = 5)	Maintaining group (n = 16)	p
Age, mean ± SD	47.5 ± 16.7	46.2±16.1	47.9 ± 17.4	0.842
Male/Female, n (%)	14 (66.7) / 7 (33.3)	2 (40) /3 (60)	12 (75) /4 (25)	0.280
Body mass index, mean ± SD	27.1 ± 3.7	27.6 ± 3.0	27.0 ± 4.0	0.333
Disease duration, years, mean ± SD	18.9 ± 17.1	12.6±9.9	20.8±18.7	0.398
Psoriatic arthritis, n (%)	7 (33.3)	3 (60)	4 (25)	0.280
PASI scores with standard dose of Risankizumab, mean ± SD (range)				
Baseline	14.3±6.2 (7.7–38.6)	11.3±1.4 (9.2–13.1)	15.3±6.9 (7.7–38.6)	0.062
Week 16	0.8±1.2 (0.0–3.6)	0.8±1.2 (0.0–2.8)	0.9±1.2 (0.0–3.6)	0.968
Week 52	0.4±0.8 (0.0–2.4)	0.5±1.1 (0.0–2.4)	0.4±0.7 (0.0–2.4)	1.000
Timing of dose reduction, weeks, mean ± SD (range)	75.4±16.7 (52–100)	80.8±16.1 (52–88)	73.8±17.1 (52–100)	0.445
PASI scores after reduced dose of Risankizumab, mean ± SD (range)				
Baseline	0.6±0.8 (0.0–2.4)	0.6±0.9 (0.0–2.0)	0.6±0.8 (0.0–2.4)	0.842
Week 12	0.7±1.1 (0.0–3.8)	1.3±1.8 (0.0–3.8)	0.5±0.8 (0.0–2.7)	0.495
Week 24	0.6±1.2 (0.0–4.4)	1.0±1.9 (0.0–4.4)	0.5±0.9 (0.0–3.0)	0.780

SD, Standard Deviation; PASI, Psoriasis Area and Severity Index.

Following dose reductions, loss of efficacy was observed in five patients. Two patients developed mild skin lesions that increased the absolute PASI score above 3 at weeks-12 and -24. Three patients had exacerbations of psoriatic arthritis at weeks-12 (n = 1) and 24 (n = 2), despite PASI scores of 0.

No significant differences were found between patients who sustained efficacy and those who did not in terms of age (p = 0.842), gender (p = 0.280), BMI (p = 0.333), disease duration (p = 0.398), or previous biologic experience (p = 1.00). The mean PASI scores at baseline were also similar (p = 0.062). None of the 10 patients who achieved a PASI score of 0 until dose reductions experienced relapses in skin lesions.

Of the five patients with loss of efficacy, standard maintenance dose treatment (150 mg every 12-weeks) was reinstituted in four. In one patient, who had experienced arthritis flares, the treatment was switched to certolizumab due to plans for pregnancy. Following dose elevations, remission was re-achieved in three patients. In one patient, although the PASI score could not be reduced below 3, the patient continued taking risankizumab because of maintenance of a PASI-75 response.

There has been growing interest in reducing biologic doses following remission of psoriatic manifestations without compromising efficacy and sustainability, with varying success rates. These variations may be partly due to differences in criteria for initiating dose reductions, dose reduction protocols, and study designs.^3^, ^4^, ^6^, ^7^, ^8^, ^9^, ^10^, ^11^ Eligibility criteria for dose reductions typically included a minimum duration of standard treatment (3- to 12-months) and achievement of stable minimal disease activity for 6 weeks to 12-months, as defined by one or more of the following criteria: an absolute PASI score of ≤3, ≤5, or ≤8; relative PASI improvements (PASI-75, PASI-90, or PASI-100); or a Physicians Global Assessment (PGA) score of 0 or 1.^3^, ^4^, ^6^, ^7^, ^8^, ^9^, ^10^, ^11^ Although most studies used relative PASI scores, we mainly utilized the absolute PASI score, as it more accurately reflects current disease severity. Absolute PASI is especially valuable in real-life settings, where baseline PASI scores are generally lower.^12^

Additionally, monitoring the maintenance of improvements following dose reductions is equally important. Most studies used the same criteria as those followed by dose reductions. This was also the case in our study. Some studies used higher absolute PASI scores (>5–8), losses in PASI 50‒80‒90‒100 responses, an increased PGA score (≥3), and/or an increased disease activity unacceptable to the patient. Variations in these criteria may make it difficult to compare treatment outcomes.

Several studies evaluated factors affecting the maintenance of remission under reduced doses. Longer disease duration, higher baseline PASI, increased BMI, and concomitant psoriatic arthritis were factors associated with failure.^4^, ^6^ Achieving PASI-100 earlier and being treated with adalimumab instead of ustekinumab and etanercept were associated with a higher success rate.^3^, ^11^ In our study, gender, age, BMI, disease duration, PASI scores at baseline and at the time of dose reductions, and coexisting psoriatic arthritis did not predict dose reduction success.

In a randomized controlled trial, patients who achieved PASI-100 response at weeks 20‒28 with guselkumab were assigned to receive either the standard every 8-week dosing or a prolonged dosing interval of every 16-weeks.^8^ By week 68, the two groups were similar in terms of disease control (PASI < 3) and levels of IL-17A, IL-17F, IL-22, and tissue-resident CD8-positive memory cells. These findings are of particular value as they demonstrate clinical and immunological disease control following prolongation of dosing intervals in patients with complete skin clearance with guselkumab. In our study, excellent skin response was maintained in all 10 patients after dose reductions of risankizumab following the achievement of a PASI-100 response.

The main limitations of our study include its retrospective design, the limited sample size, and the lack of a control group with similar patient characteristics. Nevertheless, our findings merit consideration as they demonstrate the feasibility of dose reductions following complete clearance of skin lesions in real-world risankizumab treatment. Prospective studies and randomized controlled trials are needed with larger patient populations.

In conclusion, following the achievement of low disease activity, reduced dose risankizumab maintained remission for at least 24-weeks in 76.2% of our patients. Of note, all patients who achieved complete skin clearance with standard risankizumab treatment maintained PASI-100 status following dose reduction, suggesting that the most suitable candidates for reliable and safe dose reduction are those achieving PASI-100 response. On the other hand, the higher incidence of adverse outcomes following dose reductions in patients with psoriatic arthritis warrants caution when considering dose reductions.

## ORCID ID

Tuğba Özkök Akbulut: 0000-0001-9995-2543

Algün Polat Ekinci: 0000-0003-4056-8402

## Data availability statement

The entire dataset supporting the results of this study was published in this article.

## Financial support

None declared.

## Authors' contributions

Gizem Pehlivan Ulutaş: Approval of the final version of the manuscript; critical literature review; data collection, analysis and interpretation; effective participation in research orientation; intellectual participation in propaedeutic and/or therapeutic management of studied cases; manuscript critical review; preparation and writing of the manuscript; statistical analysis; study conception and planning.

Tuğba Özkök Akbulut: Approval of the final version of the manuscript; critical literature review; data collection, analysis and interpretation; intellectual participation in propaedeutic and/or therapeutic management of studied cases; manuscript critical review.

Algün Polat Ekinci: Approval of the final version of the manuscript; critical literature review; effective participation in research orientation; manuscript critical review; preparation and writing of the manuscript; study conception and planning.

## Research data availability

The entire dataset supporting the results of this study was published in this article.

## Conflicts of interest

None declared.
